# Wham: Identifying Structural Variants of Biological Consequence

**DOI:** 10.1371/journal.pcbi.1004572

**Published:** 2015-12-01

**Authors:** Zev N. Kronenberg, Edward J. Osborne, Kelsey R. Cone, Brett J. Kennedy, Eric T. Domyan, Michael D. Shapiro, Nels C. Elde, Mark Yandell

**Affiliations:** 1 Department of Human Genetics, Eccles Institute of Human Genetics, University of Utah, Salt Lake City, Utah, United States of America; 2 Utah Center for Genetic Discovery, University of Utah, Salt Lake City, Utah, United States of America; 3 Department of Biology, University of Utah, Salt Lake City, Utah, United States of America; UCSD, UNITED STATES

## Abstract

Existing methods for identifying structural variants (SVs) from short read datasets are inaccurate. This complicates disease-gene identification and efforts to understand the consequences of genetic variation. In response, we have created Wham (Whole-genome Alignment Metrics) to provide a single, integrated framework for both structural variant calling and association testing, thereby bypassing many of the difficulties that currently frustrate attempts to employ SVs in association testing. Here we describe Wham, benchmark it against three other widely used SV identification tools–Lumpy, Delly and SoftSearch–and demonstrate Wham’s ability to identify and associate SVs with phenotypes using data from humans, domestic pigeons, and vaccinia virus. Wham and all associated software are covered under the MIT License and can be freely downloaded from github (https://github.com/zeeev/wham), with documentation on a wiki (http://zeeev.github.io/wham/). For community support please post questions to https://www.biostars.org/.

This is PLOS Computational Biology software paper.

## Introduction

Structural variation (SV) is a major source of phenotypic diversity [1–4] and human disease [5–7]. Unfortunately, detecting SVs in short-read sequence data is challenging [8]. Moreover, using SVs in association studies remains problematic, primarily due to three technical difficulties. First, SV callers suffer from both high false positive and false negative rates [5]. Second, the breakpoints of SVs are highly variable, making it difficult to detect an association between a phenotype and a complex ensemble of overlapping SVs [9]. Lastly, to our knowledge, no existing structural variant detection software can identify SV enrichment in cases vs. controls within a framework amenable to high-throughput sequence analysis. As we demonstrate, Wham (**Wh**ole-genome **A**lignment **M**etrics) effectively addresses these problems.

Current mapping based algorithms [10–16] use various attributes such as read depth (RD), paired-end mapping (PEM), split-read mapping (SRM), and soft-clipping to identify SVs. Tools that incorporate more than one of the short-read mapping signals, like Lumpy, Delly and GASVPro, show improvements over their predecessors that only use a single attribute to discover SVs [10,11,14,15]. SV callers have varying accuracy for different classes of SVs, and some have specifically designed heuristics for the identification of certain SV types. Because of this, ensemble methods, such as iSVP, SVmerge, and bcbbio-nextgen, have emerged. These methods integrate SV calls from multiple tools to improve accuracy [17–19].

Other approaches for identifying structural variants use sequence assembly methods in order to pinpoint SVs. There are two main assembly-based methods for SV detection: *de-novo* and local. *De-novo* assembly can identify SVs with great accuracy [20], but also can be prohibitively expensive in computational terms. There are also post-processing barriers for examining SVs from multiple individuals using *de-novo* assembly. For example, synchronizing the coordinates of SVs present from *de-novo* assemblies across many individuals is not a trivial task. Multiple sequence alignments provide one approach, but this is computationally expensive and is itself subject to systematic errors [21]. Another option for assembly-based SV detection is local assembly. This approach uses read mapping information to confine assembly to putative breakpoints within a genomic range, thus circumventing the need for whole genome assembly [22–24]. One drawback of local assembly is that it cannot discover large novel insertions, which might only be revealed by *de-novo* assembly, and alignment of reads to a reference genome remains problematic. Lastly, gains made possible by local and *de-novo* assembly are dependent upon higher read depths. Given finite resources, sequencing fewer individuals at a higher depth compromises power for conducting downstream association testing [25,26].

Wham’s joint SV identification and genotyping algorithms are tuned for association testing. As we show, Wham is able to pinpoint SVs in pooled and genotypic data associated with phenotypic variation. Wham thus fills the need for a fast, easy to use SV caller and association-testing tool that is compatible with most standard variant calling pipelines.

## Design and Implementation

### Identification of breakpoints and genotyping

Wham is designed for paired-end Illumina libraries with standard insert sizes (~300bp-500bp). Wham integrates mate-pair mapping, split read mapping, soft-clipping, alternative alignment and consensus sequence based evidence to predict SV breakpoints with single-nucleotide accuracy. Wham generates a combined pileup (catalog of reads covering a position of the genome) for all BAM files provided. Reads from all individuals included in joint calling that are soft or hard clipped are hashed by position to identify shared breakpoints. Positions in the pileup where three or more primary reads share the same breakpoint are interrogated as a putative SV. The soft-clipped sequences that overhang the breakpoint are collapsed into a consensus sequence using a multiple sequence alignment (MSA) provided in the seqAn library [27]. Wham applies three filters to the consensus sequences. Breakpoints are not reported in cases where consensus sequences are shorter than 10 bp or contain more than 50% mismatches in the alignment, as they more likely reflect mapping errors rather than allelic heterogeneity. Overlapping alleles that do not share the same breakpoints are reported as independent records in the VCF file, allowing for allelic heterogeneity. Different alleles with the same breakpoints that fail the mismatch consensus filter are discarded.

Wham uses split-read (SR) alignments, mate-pair (MP) positional information, and alternative alignments to find the other SV breakpoint (the breakpoint not present in the initial pileup position). Wham is unaware of past SV calls, therefore it outputs an SV call for the 5’ and 3’ breaks independently. Each split read entry in a BAM file reports the other supplemental alignments in the “SA” tag and alternative alignments are reported in the “XA” tag. Wham processes the cigar strings of the SA and XA tags to identify shared positions as candidate endpoints of the reported SV. Wham clusters all the candidate breakpoints and rounds their positions to the nearest tenth base pair. The position with the highest read support is reported. If the soft-clipped consensus sequence can be aligned to the putative breakpoint region using the Smith-Waterman algorithm, the breakpoint is further refined to the location of the consensus sequence alignment [28]. The amount of support for the breakpoint is listed in the “SP” info field.

Translocations and structural variants greater than 1 Mb undergo additional filtering. These classes of SVs can be highly deleterious genomic aberrations; therefore we require them to have additional support. Large intra-chromosomal SVs require that the other breakpoint (outside pileup position) have at least two reads supporting the exact breakpoint. This same filter is applied to putative translocations. Additionally, in the case of translocations, if the split reads in the pileup map to more than three different chromosomes, the SV is discarded. This filter removes many false positive SV calls resulting from inter-chromosomal mapping errors introduced by repetitive sequences.

Genotyping is accomplished using a bi-allelic likelihood model [29,30]. Rather than using base quality at the breakpoint position, we use the mapping quality of the read. Each read that contains the breakpoint, internally or soft clipped, is counted as non-reference. Additionally, reads that are discordantly mapped or show signs of an inversion (same strand mate pair mapping) are also considered to be non-reference for use in genotype calling. During joint calling at least one individual must have three reads supporting the alternative allele. This filter prevents randomly shared start and stop soft clipping across individuals from triggering a non-reference allele call.

For best performance, we recommend using BWA mem [31] followed by sorting and duplicate removal of the BAM files (duplicate marking is also supported). The BWA mem algorithm provides soft clipping and split read annotations. Specifically the “SA” and “XA” optional fields in the BAM files are heavily utilized by Wham. Supplementary read alignments (0x800 / split reads) can be marked as secondary with no detrimental effect. Marking or removing duplicates is highly recommended as these duplicates cause false positive SV calls. Other mapping software like Bowtie2 [32] provides soft clipping, which is sufficient to run Wham, but not recommended. Wham can be run on single-end sequencing data, but for best results, paired-end data are recommended.

### Classification of SV type

Wham classifies the type of structural variant using a random forest of decision trees implemented in scikit-learn [33]. This approach is similar to another SV caller, forestSV [34]. Wham’s raw breakpoint calls (in VCF format) are post processed by ‘classify_WHAM_vcf.py’ to add SV type to the INFO field. The wham classifier provides the SV type in the “WC” info field and probability of each type in the “WP” info field. We use fourteen attributes of a genomic position for the classifier (S1 Table). Each attribute is a fractional measure reflecting the number of reads that belong to each attribute, normalized by the read depth at the pileup position. Some of the fourteen attributes have low to no importance for training the model, but we chose to maintain them as they allow further downstream development. The training dataset is derived from our simulated dataset, which includes deletions, insertions/translocations, duplications and inversions. The k-fold cross-validation implemented in scikit-learn reports a validation rate of ~0.94 for the simulated dataset. Users may create their own training set consisting of a truth set of variants, supplying as many variant types as they see fit. To do this, Wham should be run over a BAM file containing SVs that have been validated. Then the “AT” info field should be split into a tab-delimited file with the last column providing the validated SV type. The resulting training file should match the format of the file distributed with Wham. Additionally, Wham can be extended to annotate as many features as the user sees fit. False positive Wham SV calls can also be annotated and added to a training set. This flexibility makes Wham extendable to identify many patterns in a pileup that differentiate between SV types.

### Wham’s association test

When the “target” and “background” options are enabled, Wham quantifies the difference between the target and background allele frequencies using a likelihood ratio test (LRT) under a binomial likelihood model with one degree of freedom. The basic LRT used within Wham has been widely adopted for association studies [26,29,35]. However, the LRT is intended as a simple first-pass association test for Mendelian traits; it cannot account for population stratification and relatedness between individuals and is not well suited for quantitative traits. For more robust association tests, we recommend analyzing Wham’s genotypes with tools such as PLINK or TASSEL [36,37]. Wham’s LRT has also been implemented in GPAT++, a population genetics library [38].

The null model of Wham’s LRT assumes that the allele frequencies of both the target (*AF*_*T*_) and background (*AF*_*B*_) groups have the same distribution, while the alternative hypothesis is that the allele frequencies of the two groups come from two separate distributions. The allelic counts in the model come from the genotype calls.

$$D=-2\;*\;ln(\frac{B(N_{C},K_{C},{AF}_{C})}{B(\;N_{T},K_{T},{AF}_{T}\;)\;\times \;B(N_{B},K_{B},AF_{B})})$$

Where:

The binomial density function (B*(n*, *k*, *p*)) is parameterized by the number of successes *n*, the number of trials *k*, and the probability of success *p*. In the current application, *n* is the number of non-reference alleles in the target (N_T_), background (N_B_) and the target/background combined (N_C_). The parameter *k* is the number of alleles in the target (K_T_), background (K_B_) and the target/background combined (K_C_). The probability of success, *p*, corresponds to the target (AF_T_), background (AF_B_) and combined (AF_C_) allele frequencies. Wham reports the *D* statistic in the “LRT” info field. Larger LRT values can indicate that the null hypothesis should be rejected under the assumptions of the binomial model. A chi-squared lookup, with 1 df, can be used to convert the *D* statistic into a p-value.

For instructions regarding the installation and use of Wham, refer to the wiki-page (http://zeeev.github.io/wham/).

## Results

Wham integrates multiple mapping-based signals to identify putative SV breakpoints. Both individual genome and populations of individuals (pooled sequencing) data sets can be processed with Wham. Additionally, if two cohorts of genomes are provided (target and background), Wham can be used to conduct an association test. This provides means both to identify SVs with genotype-phenotype associations and to filter SV false positives. Wham also classifies types of SV (deletions, duplications, inter-chromosomal events/insertions, and inversions). Classification is performed *post hoc*, as Wham conducts genotyping and association testing independent of the SV type. Here we explore the accuracy of Wham’s SV detection and genotyping, first by using simulated short-read datasets, followed by two whole genome human datasets. We also use Wham to identify biologically important structural variants in non-human data.

For a detailed description of the datasets used for the following analyses and software versions see the Supporting information.

### Validation of Wham using simulated data

We first examined the performance of Wham’s SV detection heuristic and compared it to three other SV callers, Delly [11], Lumpy [14] and SoftSearch [15], using simulated whole genome sequencing (WGS) data. Synthetic reads were generated for 10x and 50x whole genome coverage with simulated occurrences of four classes of structural variants (deletions, duplications, inter-chromosomal events/insertions, and inversions; see Supporting Information for details). Simulated insertion events were created by placing sequences from other chromosomes into alternate locations mimicking inter-chromosomal copy number variants; we will refer to these events as insertions throughout the rest of this section. We chose to benchmark Delly, Lumpy and SoftSearch because all three tools can identify multiple types of SVs, are widely used, and are easy to install and run directly from BAM files. Lumpy also provides a point of reference against GASVPro and Pindel, as it has already been benchmarked against these tools under matched simulation conditions [10,39]. We used a previously published interval size, (regions defined by 25 bp up- and downstream of each simulated variant breakpoint) as “truth intervals” to determine true positive calls, unless otherwise noted [14]. A SV is considered a true positive only if both of the called breakpoints lie within a “truth interval.” For specific details regarding the simulations see Supporting Information.

**Fig 1:**
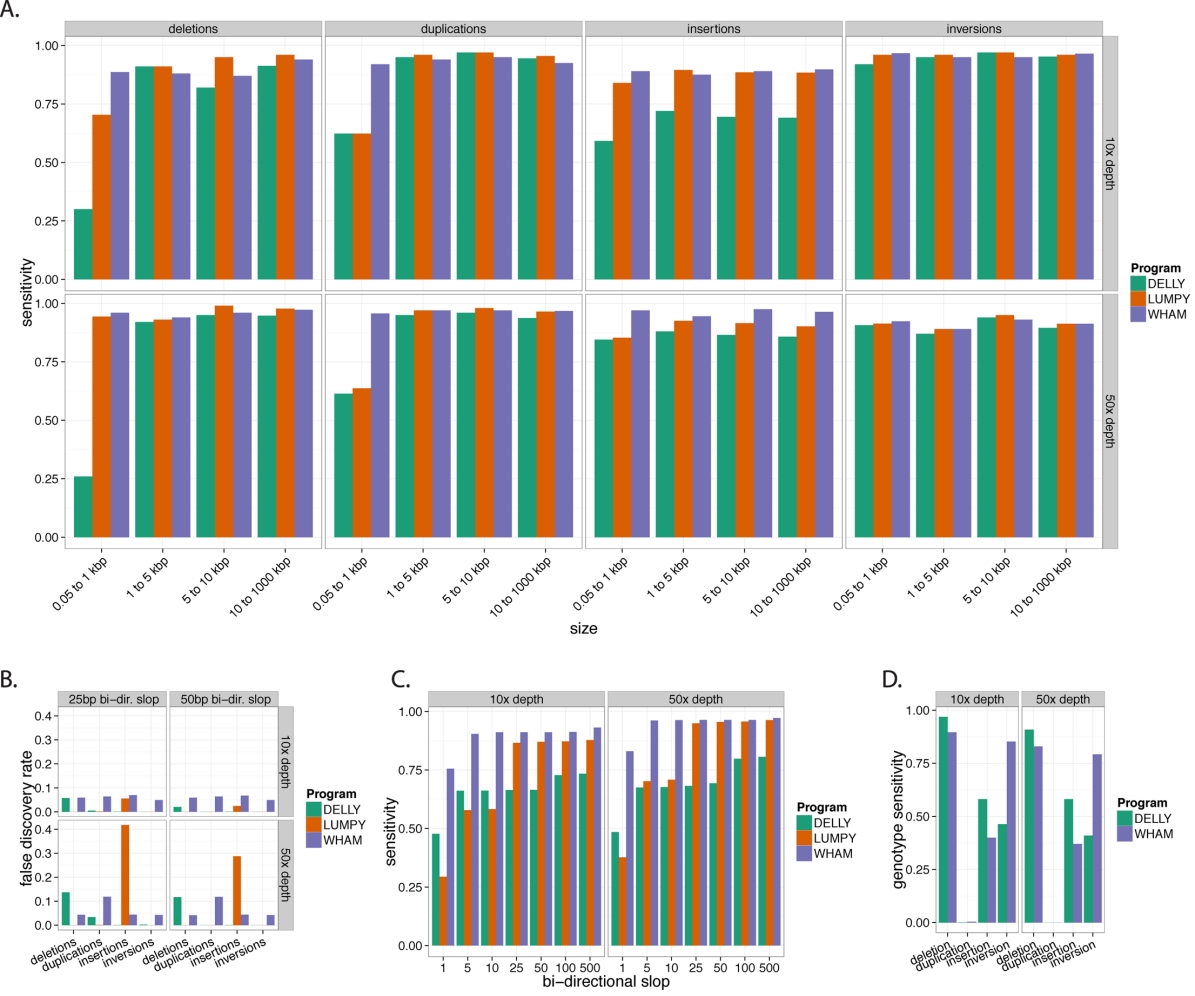
Sensitivity and false discovery rates (FDR) for simulated data. The sensitivity and FDR of Delly, Lumpy, SoftSearch and Wham for simulated deletions, duplications, insertions and inversions. The sensitivity is measured for each category at depths of 10x and 50x. SVs ranging from 50 bp to 1 Mb are grouped into four left-closed size intervals. **A)** The sensitivity of the three tools is faceted on size, depth and SV type. At 10x Wham has noticeably better sensitivity for deletions and duplications in the smallest size class. Wham’s sensitivity is higher than Delly and Lumpy for insertions at 10x and gains sensitivity at 50x. **B)** The FDR for each type of SV faceted by depth and the amount of slop added to each confidence interval. In the 25 bp slop category, each confidence interval was extended in both directions by 25 bp. At 10x depth Wham has the highest FDR across all SV classes and Lumpy has the lowest. At 50x Delly has heightened FDR for deletions and Lumpy has a much higher FDR for insertions. Shrinking the confidence intervals increases the FDR for Delly and Lumpy, but not Wham. **C)** Breakpoint sensitivity for deletions. The confidence intervals, provided by the three tools are ignored and slop is incrementally added to the predicted breakpoints. Wham has the highest sensitivity when 1–10 bp of slop is added. **D)** Genotype sensitivity for the homozygous non-reference simulated SVs. Delly and Wham have similar sensitivity for deletions and duplications while both tools fail to correctly genotype duplications.

Wham, Lumpy, Delly, and Softsearch were run in their default modes across the simulated data to identify SV breakpoints. For the high-depth simulated dataset (50x), Wham and Lumpy have comparable sensitivity overall (0.94 and 0.90 respectively), while Delly has slightly lower sensitivity (0.84). Softsearch had the lowest sensitivity for the simulated dataset (0.74). The structural variant size drives the largest differences in sensitivity between the three tools (Fig 1A). For example, Lumpy, Delly and Softsearch are not able to detect many of the smaller duplications (50 bp and 100 bp; collapsed into the 0.05–1 kb interval [Fig 1A]). Delly’s limitation in detecting small SVs (~300 bp) has been acknowledged by the authors. For smaller SVs (<60 bp) the sensitivity of Wham is generally 2–3 times greater than the other tools (Fig 1A; 0.05–1 kb interval). All three tools have similar sensitivity for detecting simulated SVs greater than 1 kb in the 50x dataset. Given that the observed frequency of SVs follow a power law distribution with respect to size, we expect that Wham will discover more SVs on real biological datasets than the other tools [40,41]. Compared to its performance on other classes, Wham has the lowest sensitivity for insertions in the 10x coverage simulated dataset (Fig 1A). This is due to Wham incorrectly identifying one of the two breakpoints at lower depths, which ceases to be a limitation at higher depth of coverage. These sensitivity assays demonstrate that Wham excels at finding small SVs (less than 1 kb) while maintaining similar performance to the other tools for SVs greater than 1 kb.

Next we assayed the false discovery rate (FDR) of the four tools on the same simulated data. Wham has an FDR of 0.05 at 50x with 50 bp of slop (see Supporting Information), which is higher than Delly (0.02), but lower than Lumpy (0.11) and SoftSearch (0.41) (Fig 1B). Lumpy has the lowest overall FDR if insertions are excluded. Reducing the amount of slop added to the confidence intervals slightly increases the FDR for Delly and Lumpy, but not for Wham or SoftSearch. Wham’s FDR can be attributed to misclassification of SV type and failures when identifying both breakpoints. All three tools exhibited a positive correlation between depth and FDR when comparing the 10x and 50x datasets. For example, Delly’s FDR for deletions nearly doubles in the 50x relative to the 10x data. All three tools had elevated FDRs for insertion events. This is because our simulated insertions create inter-chromosomal duplications which increase mapping errors leading to false positive SV calls. As expected, the FDRs for the simulated data are much lower than the human benchmarks, as discussed below. The change in FDR between simulated and experimental data can be attributed to the simplicity of the simulations. For example, we did not model errors in the reference genome or mobile element insertions, which would have increased the baseline FDR for all the tools.

To assess the breakpoint accuracy of the tools, we removed the confidence intervals for deletions and then incrementally added 1–500 bp of bi-directional slop to both breakpoints (Fig 1C). Wham has the highest positional accuracy for deletions of the four tools, as it has the highest sensitivity (0.75) with only 1 bp of slop. Lumpy exhibits a marked gain in sensitivity from 10 bp to 25 bp of slop as it is designed to detect “soft” breakpoint boundaries [14], whereas Delly’s sensitivity exhibits an increase from 1 bp to 5 bp of slop. In contrast, Wham maintains a near constant sensitivity down to 5 bp of slop, after which Wham’s sensitivity drops, but remains greater than 0.75. Wham maintains sensitivity at small intervals by relying on highly accurate mapping and soft clipping. Wham and SoftSearch, unlike the other tools, use soft-clipping information to call small SVs. However, Wham loses less sensitivity from 5 bp to 1 bp than Softsearch. Wham’s breakpoint sensitivity is important for maintaining power during association testing. The power to detect an association between a SV and a phenotype is diminished when breakpoints are miscalled within a cohort of affected individuals. All four tools showed improved breakpoint detection at higher depth (Fig 1C). The high positional sensitivity shows that mapping-based methods can reliably localize SV breakpoints down to a 3-bp interval. This small interval provides sufficient accuracy for association testing.

Collectively, these simulations show that Wham provides a robust means for SV identification. Compared to the other three tools, Wham excels at finding smaller structural variants across all simulated SV classes and has the highest breakpoint sensitivity. This is important as SVs are distributed geometrically with respect to length, and thus shorter SVs comprise the vast majority of real events [42,43]. As we show below, Wham also maintains high sensitivity when using real human short-read data, but at the cost of a much higher false discovery rate.

### Validation using human WGS data

For our human benchmarks, we started with NA12878, the best characterized human genome. For the truth set we used the 2,597 NA12878 non-reference genotype calls in the 1000 Genomes SV dataset (phase III submitted calls) [42], downloaded from dbVar (estd214) [43]. This dataset contains SVs ranging in size from ~200 bp to ~900 kb. Deletions, large indels and mobile element insertions make up the majority of the NA12878 subset. It is worth noting that Delly calls are represented in this truth set, but calls from Lumpy, SoftSearch and Wham are not. We ran each SV caller, as per best practices, over NA12878 generating between ~10K and 400K SV calls (S2 Table). The expected number of SV calls in NA12878 depends on the SV size range and the tolerated FDR. The 1000 Genomes Project imposes a 5% FDR for structural variants, improving the accuracy at the expense of sensitivity [42]. Therefore the 1000 Genomes Project SV calls for NA12878, while highly accurate, are an underestimation of the total number of SVs for this genome. In stark contrast, another group (Bickhart et al. 2015) reported over a million deletions in NA12878 [44]. The high number of SV calls for NA12878 made by Wham, reported in S2 Table, and reported by Bickhart et al. highlights the importance of *post hoc* filtering prior to benchmarking against a truth set. S2 Table lists the three filters we used to improve both the sensitivity and FDR of the tools benchmarked here. After all filtering, Wham, Delly, SoftSearch and Lumpy had 84.4k, 2.2K, 25.3k, and 3.6k SV calls, respectively (S2 Table).

**Fig 2:**
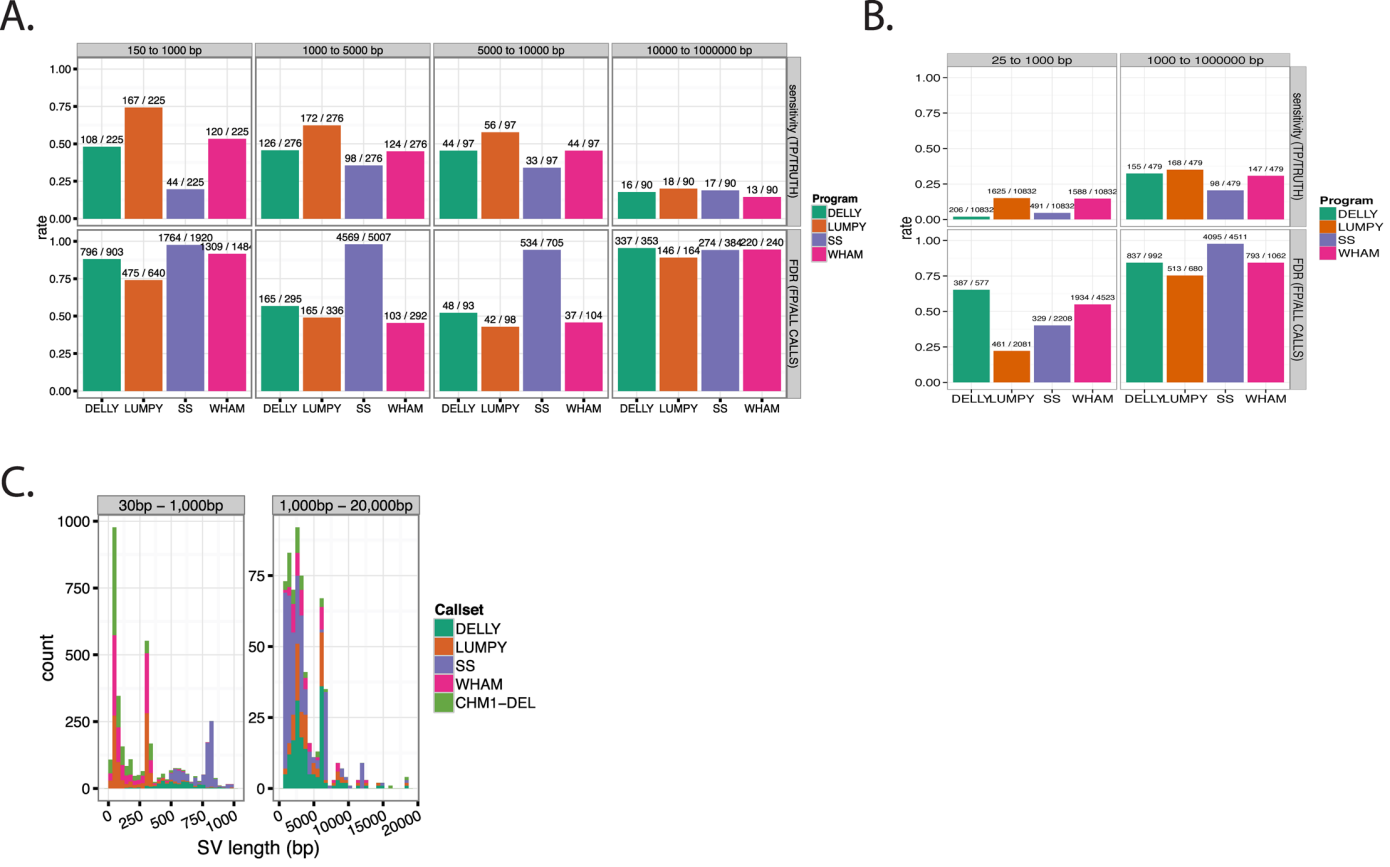
Benchmarking Delly, Lumpy, SoftSearch and Wham against NA12878 and CHM1 datasets. **A)** The sensitivity and FDR for filtered NA12878 Phase III deletion calls across four size intervals. The number of true positives and the number NA12878 calls are listed above sensitivity, while the total number of false positives and total calls for each tool is listed above FDR. Most true positives and false positives are within the 150–1,000 bp interval. **B)** The sensitivity and FDR for CHM1 deletions. **C)** The size distribution of the true positive calls that overlap the CHM1 deletions. One thousand true positives were randomly sampled from each tool and the truth set (CHM1-DEL).

Lumpy has the highest sensitivity (0.60) and lowest FDR (0.66) overall for NA12878 deletions. The sensitivity for all three tools starts at ~0.5–0.75 in the 150 bp to 1 kb interval and tapers off to ~0.25 for SVs greater than 10 kb (Fig 2A). The FDR was high for the smallest and largest size categories (100 bp to 1 kb and 10 kb to 1 Mb). Between these two categories, the smallest size variant window contained the largest number of false positives and true positives for each tool. For example, Delly has over 108 true positives and 769 false positives in the smallest size category. Wham’s sensitivity (0.43) was lower than Lumpy’s (0.66), but not Delly’s (0.42) or SoftSearch’s (0.28), and overall Wham’s FDR (0.78) was below those of Delly (0.81) and SoftSearch (0.89). The two methods that rely on soft-clipping (SoftSearch and Wham) had higher numbers of calls compared to the other methods. Wham emits a call for each breakpoint, and therefore Wham suffers two false positives for every SV using our benchmarking framework.

For a second, independent, human benchmarking experiment we used the recently published single-molecule, real-time (SMRT) sequencing dataset of a hydatidiform mole cell line (CHM1) [45]. The hydatidiform genome comprises a duplicated male haploid (double haploid). The PacBio SMRT SV calls are a good standard for validating Wham’s performance on the related Illumina datasets because PacBio SMRT sequencing does not require DNA cloning or amplification, two common sources of sequencing artifacts. Moreover, the absence of allelic heterogeneity in the haploid CHM1 genome facilitates accurate assembly [45,46]. Additionally, PacBio reads can capture small and moderately sized SVs internally within a read, providing a more accurate source for detecting SVs. Both PacBio and Illumina sequence data were generated from DNA recovered from CHM1 cells. The 101-bp Illumina reads and PacBio (~8 kb average length) reads cover the haploid genome to 40.7x and 36.6x depth, respectively. The structural variant calls from the PacBio single molecule sequencing were generated by first identifying putative SV breakpoints followed by local assembly (see supporting information of [45]). We analyzed the Illumina data with Wham, Delly, Lumpy and SoftSearch, and compared their SV calls to the 11,311 SMRT deletions (http://eichlerlab.gs.washington.edu/publications/chm1-structural-variation).

Wham and Lumpy have similar sensitivities for CHM1 deletions, while Delly and SoftSearch lag behind (Fig 2B). For SVs larger than 1 kb, all tools have similar sensitivity for CHM1 deletions. Overall, the sensitivity and FDR for the CHM1 dataset was lower than for the Phase III NA12878 1kg dataset. There are several possible explanations for this difference. First, the sensitivity might be lower because many of CHM1 PacBio calls are in repetitive regions that cannot be detected with Illumina short-read mappings. The FDR may be lower because the CHM1 dataset contains ~ 10 times more calls than the 1kg NA12878 dataset. Lastly, the CHM1 SV size distribution contains smaller calls than the 1kg N12878 dataset. We examined the size distribution of true positive calls by Delly, Lumpy, SoftSearch and Wham (Fig 2C). Wham’s size distribution for deletions closely tracks the CHM1 dataset. Both datasets are enriched for deletions less than 100 bp, which is concordant with previous studies [42,47]. The peaks in the size distributions at 300 bp and 6000 bp correspond to ALUs, STRs, and LINE-1 elements. The variability between the size distributions of the tools suggests that each tools is well suited for a slightly different size class.

### Genotyping accuracy

**Fig 3:**
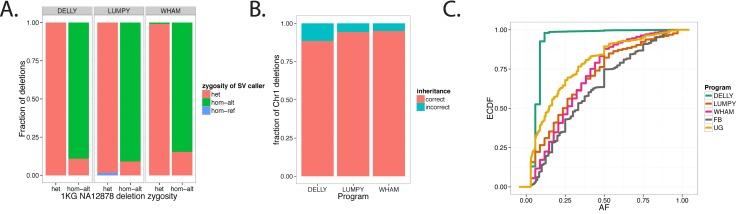
Genotyping assays. **A)** Comparison of Genome STRiP (GS) genotypes vs. Delly, Lumpy and Wham. The x-axis lists the GS genotype. Different colors denote the zygosity of the Delly, Lumpy, and Wham genotypes. **B)** The fraction of Chromosome 1 deletions for the NA12878, NA1277, and NA12882 trio that conform to Mendelian inheritance patterns. **C)** The CEPH/Utah Pedigree 1463 allele frequency (AF) spectrum represented as an empirical cumulative distribution function (ECDF). This curve is derived from Chromosome 1 deletions. FB, Freebayes; UG, Unified Genotyper [49].

We began to assay the genotype accuracy of the Delly, Lumpy (svtyper) and Wham by comparing their calls to Phase III NA12878 1kg deletions (genotyped by Genome STRiP [48]). SoftSearch was excluded from all genotyping assays because it only provides a hard-coded heterozygous genotype call. We measured how many genotypes were misclassified (Fig 3A). Delly has the highest fraction of concordant calls followed by Lumpy and Wham, however all tools differ by only a few percent. Wham and Delly tend to call homozygous non-reference genotypes as heterozygous. Lumpy, unlike the other two tools, calls a small percentage of heterozygous genotypes as homozygous reference. Wham calls a small fraction of heterozygous genotypes as homozygous non-reference.

Next we used the Platinum trio of human genomes (NA12878, NA12877 and NA12882) to measure Mendelian violations for deletions. Wham had the lowest number of Mendelian violations followed by Lumpy and Delly (Fig 3B).

To ensure that Wham provides robust multi-sample genotype calls, we calculated the allele frequency spectrum for Chromosome 1 deletions in the CEPH 1463 pedigree (Fig 3C). The CEPH 1463 pedigree has 17 family members, across three generations, with the final generation consisting of 11 siblings. Based on the structure of the pedigree, we expect to see the allele frequency spectrum skew toward common variants (Fig 3C). Both Wham and Lumpy have spectra that are similar to FreeBayes and Unified Genotyper small deletions. Delly stood out as it overcalled rare variants, even after quality filtering.

The results from benchmarking on real data have several important implications. First, Wham achieves comparable performance relative to other commonly used structural variant callers for deletions. Importantly, Wham provides robust means for discovering small structural variants. Second, the low overlap between SV classes among the tools tested here supports the power of integrated SV call sets. Frameworks, like the approaches of bcbio, which acts by combining SV calls from a variety of callers (including Wham), can capture a greater swath of genetic diversity while also providing higher confidence for concordant allele calls across varying heuristic methods [17–19].

### Identifying candidate SVs with Wham’s association test

Although Wham, Delly and Lumpy have similar sensitivities for NA12878 deletions, any critical appraisal of their performance must take into account the very high false discovery rates of all three tools. Using the NA12878 deletion data, for instance, Wham’s FDR is 0.78, Delly’s is 0.81 and Lumpy’s is 0.66. These values illustrate just how difficult SV discovery is using short read data. However, for purposes of genotype-phenotype association, high false discovery rates are tolerable so long as false positives are either randomly distributed across cases and controls (non-differential error), or are systematic (e.g. called in every individual). In both scenarios false positives will cancel out in an association test. Thus, given a reasonable true positive rate, robust association signals will be obtained even in the face of very high FDR.

We first sought to test if Wham’s non-differential false discovery rate creates spurious signals of association. To examine this, we used a cohort of individuals with a high degree of genetic relatedness such that if they were assigned randomly into two groups for association testing, there should be little to no differentiation. We chose the CEPH/Utah Pedigree 1463, comprised of seventeen individuals across three generations [42,50]. This pedigree should not harbor appreciable levels of population stratification, thus removing a potential confounding source of false positive associations in our sampling. Wham was run in default mode three times, randomly dividing the pedigree into two groups of eight individuals for assignment to either target or background groups. One genome was excluded each round so that the target and background had the same number of individuals. True and false SV calls were assigned according to their proximity to the phase III 1000 Genomes Project SV calls using a 50-bp truth interval. In total, 16,470 association tests were run for the true positive SV calls, while 380,005 were run for the false positives. Comparing the distributions of Wham’s LRT p-values (Chi-squared one degree of freedom) between the groups showed a significant difference between the true and false SVs, as shown in S1 Fig (Two-sample Kolmogorov–Smirnov [KS] test D = 0.0948, p-value = 2.2e-16). The median p-value for the true positive group was 0.66 and 0.69 for the false positive group (1.03 times higher). To see if the significant difference is robust to the number of variants assayed, we subsampled 100 Wham p-values for both groups and the KS test was re-run. Over 1000 iterations, only 362 of the 1000 KS tests achieved significance. This suggests that there is a small, albeit significant, difference between true positives and false positives. Together, this demonstrates that Wham’s false SV calls are only expected to slightly inflate the number of spurious associations.

While Wham has a high false discovery rate for SV detection, Wham’s association-testing framework is robust to many of these errors as they are non-differential between the cases and controls. To demonstrate that Wham’s high FDR for SVs does not hinder association studies we used Wham on both genotypic (pigeon) and pooled (viral) datasets. In the pigeon dataset, Wham was used to re-map a causative SV for the recessive red pigmentation trait and in the viral dataset we show that Wham reliably identifies the breakpoints of a duplication involved in viral adaptation.

### Identifying the genetic basis of recessive red coloration in domestic pigeons

Pigeon fanciers have selected for a wide range of phenotypic variation in domestic pigeons over thousands of years. These traits include plumage patterns, behavior, body size and pigmentation [51]. Several alleles in three genes–*Tryp1*, *Sox10*, and *Slc45a2* –were recently identified that produce variation in melanin synthesis [52]. For example, birds homozygous for a deletion spanning a melanocyte-specific enhancer of *Sox10* have reduced expression of *Sox10* and its target *Tyrp1*, resulting in the ‘recessive red’ color phenotype (classical *e* locus). Using a previously generated WGS dataset, we examined the power of Wham’s association test to identify the *e1* allele of recessive red, a 7.5-kb deletion on scaffold974 of the pigeon genome assembly (C_liv1.0 [53]). In conjunction with the Wham analyses, we also ran the same association test (implemented in pFst [38]) for SNPs and Delly SV genotype calls.

**Fig 4:**
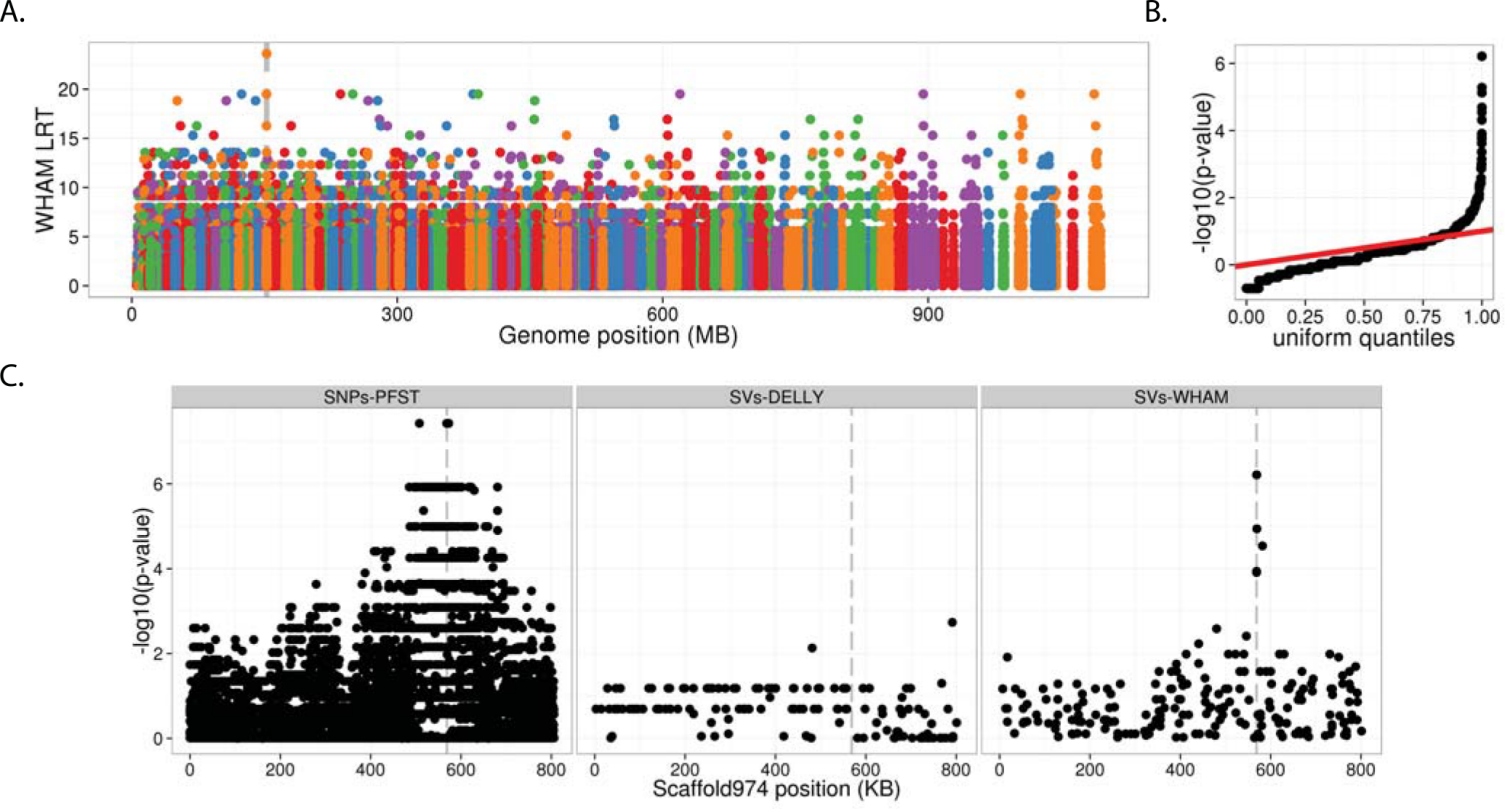
Identification of the *e1* allele using Wham’s LRT. **A)** Wham’s LRT interrogates allele frequency differences between recessive red and wild type birds. Genomic scaffolds are denoted by different colors and are sorted by size in increasing order. The highest LRT score (dashed vertical line) is a 7.5-kb deletion upstream of the *Sox10* gene, which encodes a transcription factor that is critical to the melanin synthesis pathway. Only LRT values above 1.5 are shown in A. **B)** The quantile-quantile plot after converting Wham’s likelihood ratio values to p-values. **C)** Scaffold974 association tests from SNPs, Delly SV calls and WHAM SV calls.

Wham identified the *e1* allele as the best genome-wide candidate for recessive red using a likelihood ratio test (LRT; Fig 4A, Fig 4C). The LRT implemented in Wham measures the differences in allele frequencies based on the genotype calls at every SV position in the genome. Five recessive red and six wild type birds were processed with Wham to identify SVs and conduct association testing. The highest Wham LRT scores localized at the two PCR-confirmed breakpoints of the *e1* allele on scaffold974 (Fig 4C). Because the pigeon reference genome was assembled from a recessive red bird that harbored the *e1* deletion allele, Wham indirectly identified the location of the deletion by identifying an “insertion” in the wild-type birds, relative to the reference genome. Delly was unable to identify this allele, because it was not designed to identify novel insertions (Fig 4C). Wham also detected several small inversions near the deletion breakpoints of the *e1* allele, whereas Delly additionally failed to detect these inversions. The increased LRT scores (converted to p-values) around the *e1* allele are attributable to linkage disequilibrium since *e1* is on a haplotype shared by all of the recessive red birds we tested. This linkage is even more pronounced in the SNP data, which have a much higher density of variants. The p-values from Wham’s association test fit a uniform distribution, suggesting little to no population stratification between the cases and controls in domestic pigeon (Fig 4B). Importantly, Wham’s high false discovery rate did not affect our ability to find the *e1* allele. This analysis demonstrates the utility of Wham for rapidly and confidently identifying a structural variant associated with a Mendelian trait in a non-model system.

### Identifying adaptive structural variation in vaccinia virus populations

Structural variants in the form of gene copy number variation (CNV) in DNA virus genomes provide a mechanism for rapid virus adaptation to host immune defenses [54–57]. For example, frequent recombination events creating tandem gene duplications have been observed in vaccinia virus (VACV) as a means of adaptation to the human antiviral host factor protein kinase R (PKR) during experimental evolution [55]. In this system, selective pressure was placed on the virus by deleting the E3L gene encoding a strong PKR inhibitor, leaving only a weak PKR inhibitor encoded by the K3L gene [58]. Experimental evolution of this ΔE3L virus in HeLa cells revealed that copy number expansion of the K3L gene provides gains in viral fitness. To test whether CNV is a common mechanism of adaptation and determine whether Wham is an effective tool to detect and characterize such events, we passaged the ΔE3L VACV strain ten times in a different cell line derived from primary human fibroblasts.

**Fig 5:**
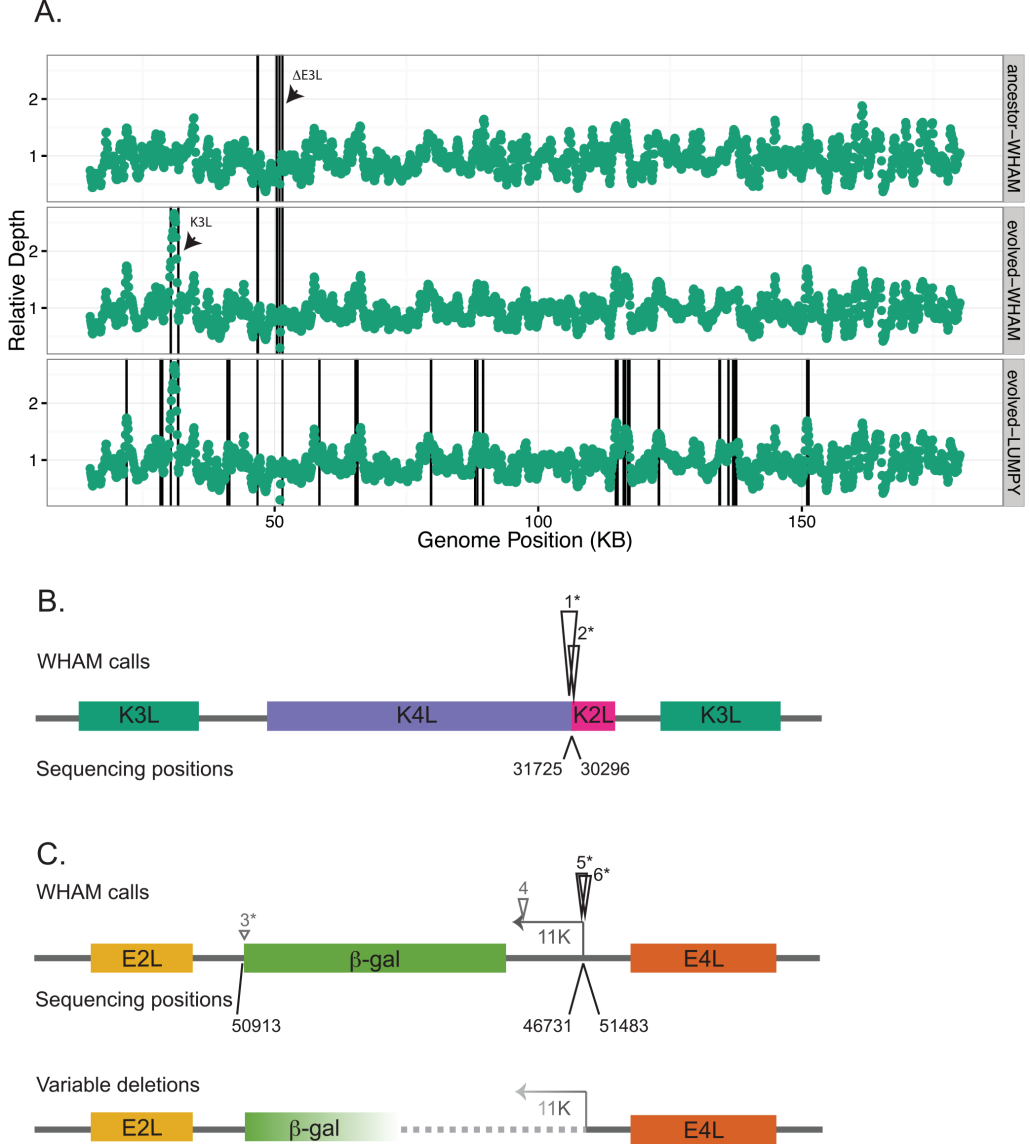
Wham detects structural variation in vaccinia virus populations. **A)** Read depth normalized within each sample is plotted across the ~200 kb vaccinia genome (excluding inverted terminal repeats) for either the parental strain (top panel) or an adapted strain (middle and bottom panels, called by Wham or Lumpy, respectively). Arrows highlight the positions of K3L CNV and E3L deletion. The black lines represent the breakpoints of every SV call after filtering (see Supporting Information). **B)** Wham calls in the adapted strain near the K3L duplication breakpoint are shown as black triangles above the viral genes in colored boxes. The height of the triangle represents split-read (SR) count supporting the call. Sanger sequencing positions relative to the reference sequence are listed below. Asterisks (*) indicate Wham calls that match the exact breakpoint determined by Sanger sequencing (see S3 Table for Wham and Lumpy breakpoints). **C)** Wham calls in the adapted strain near the E3L deletion are shown above the genes, and Sanger sequence confirmed positions below, as in B. The arrow indicates the position of the 11K promoter driving β-gal expression. For breakpoints in grey, the height of the triangle indicates the relative mate-pair count from Wham, as these positions do not have SR support.

We analyzed short-read sequencing data from viral genomes obtained from a virus population after ten passages and the parental ΔE3L strain for comparison. This analysis revealed areas of structural variation in the adapted viral population. Plotting read depth across genomic positions revealed a spike in read depth corresponding to the K3L locus that is only present in the adapted strain (Fig 5A). This is consistent with previous work in which a similarly large increase in depth corresponded to increased K3L copy number as a means of adaptation [55]. To determine the exact position of the recombination event generating the CNV, and to find any novel structural variants, we performed SV calling using either Wham or Lumpy. Using similar filtering schemes, Wham identified 6 SV calls in the adapted viral population, compared to 20 SV calls identified by Lumpy. Overlaying SV breakpoint calls on the read depth plots shows the increased specificity of using Wham to identify SVs (Fig 5A).

Wham analysis identified four SVs in the parental strain, and an additional two in the adapted strain. Notably, all six SVs map near the K3L locus or the E3L deletion (Fig 5B, S3 Table). The two breakpoints near the K3L locus were only identified in the adapted population, suggesting that the SV was not present in the parental strain. These two breakpoints have very high read support, indicative of the same recombination event dominating throughout the adapted viral population (S3 Table). Indeed, when we specifically amplified and sequenced the region around the K3L breakpoint, we identified a single breakpoint in the adapted strain, but could not detect any SV in the parental strain at this location. Importantly, the Wham-identified breakpoints match the exact positions of the breakpoint identified by PCR and Sanger sequencing (Fig 5B). Thus Wham is able to identify SVs in viral populations, down to single nucleotide accuracy. This analysis also suggests that K3L CNV is a common mechanism for VACV to overcome the antiviral PKR defense pathway.

Surprisingly, the other four breakpoints Wham identified with high read support map near the E3L deletion (Fig 5C and S3 Table). This is unexpected because E3L was originally replaced with a β-galactosidase (β-gal) selective marker, creating an insertion much larger than the reads from deep sequencing. However, the ΔE3L virus was originally engineered to express β-gal under the control of the VACV 11K promoter. This promoter naturally drives expression of the F17R gene, and is thus present in the reference genome at the F17R locus approximately 5 kb upstream of E3L. Therefore, one end of the E3L deletion is supported by split reads mapping to the natural viral promoter. The other end of the deletion does not have SR support as the β-gal gene itself is not in the reference genome, but Wham did pick up a breakpoint with mate-pair support at this end. Importantly, the genomic positions identified by direct sequencing of both the parental and adapted strains for each end of the E3L deletion were correctly called by Wham (Fig 5C). These results show that Wham can identify both a genetic rearrangement (K3L) and a novel insertion (β-gal) with respect to a reference sequence. In comparison, while Lumpy successfully identified the K3L duplications (also down to the exact base pair on one end), it failed to identify two of the E3L breakpoints detected by Wham. Overall, three of the five breakpoint positions identified by direct sequencing were called using Lumpy, although the remaining two are in close proximity to one of the called positions. Also, only one of these positions exactly matches the sequenced position, consistent with Lumpy providing a region rather than a specific position. Thus, in this experiment, Wham shows greater specificity as demonstrated by fewer total SV calls, as well as improved accuracy when compared to Lumpy in analyzing this data set. While Wham’s low call rate in this example is not consistent with the human data, there are two possible explanations for this trend: the truth sets for the human data are under-called resulting in Wham’s high FDR, or Wham under calls pooled datasets. The second possibility is unlikely since Wham correctly identified the breakpoints of all SVs independently verified in the viral dataset [55].

Taking a closer look at Wham calls with mate-pair (MP) support in addition to SR calls, we discovered a complex set of breakpoints around one end of the E3L deletion. For the K3L breakpoint, Wham only called the two positions of the single breakpoint, whereas it called one additional position with high read support on one end of the E3L deletion (Fig 5B and 5C). To determine whether these calls represent true variants, we performed PCR and Sanger sequencing across the region spanning from E2L to E4L, which includes the entire β-gal cassette. This analysis revealed SVs in both the parental and adapted strains that contain partial deletions of the β-gal and the 11K promoter. Thus Wham correctly identified a previously unknown variable deletion (Fig 5C). We have two hypotheses to explain the appearance of variable deletions in this region. First, in the absence of selection on the β-gal marker gene, there is a fitness cost to carrying the engineered marker, so viruses losing this region have a fitness advantage compared to ones retaining it. Alternatively, using a VACV promoter for β-gal expression present at a second location only ~5 kb away in the genome might promote localized recombination in this region of the VACV genome. These hypotheses are not mutually exclusive and highlight how genetically engineered virus strains may not always be homogenous.

In addition to identification of SVs from sequencing individual genomes, this analysis demonstrates that Wham is able to detect variable structural changes within polymorphic populations. This provides an example of Wham’s utility as a tool for accurate detection of SVs in rapidly changing microbial populations. Gene amplification can play a major adaptive role in response to selective pressure in both viral [54–57] and bacterial populations (reviewed in [59–62]), so it is important to accurately define the adaptive potential of structural variants. Recent advances in whole genome sequencing provide a wealth of genetic information about microbial population dynamics, and Wham provides a tool to rapidly identify potentially adaptive SVs.

### CPU usage, memory and runtime

Of the four tools used in the benchmarks, Lumpy has the fastest runtime and lowest memory requirements, but with two important caveats. First, the preprocessing steps require reading through each BAM file at least once. Second, Lumpy’s genotyper, SVtyper, took three days to run with one CPU for NA12878 deletions compared to less than a day for Wham or Delly. Filtering out high coverage regions drastically improved SVtypers performance. SoftSearch was prohibitively slow in the human benchmarks. To successfully run SoftSearch we split the BAM files into smaller genomic regions. Delly and Wham do not have filtering steps and joint call samples, thereby allowing a more direct speed comparison (S2 Fig). Wham’s runtimes increase linearly with the number of samples. Wham’s memory requirements depend on the number of CPUs (threading), the number of individuals, and the read depth since Wham maintains a pileup for each individual. Wham’s performance and memory usage is similar to other widely used SNP calling tools.

### Conclusions

Wham is a flexible SV caller that works on a broad range of data including pooled and diploid individuals. Wham’s SV detection compares favorably with other popular mapping based SV calling methods and performs well across a number of SV types in both simulated and real datasets. Like other SV calling tools, Wham suffers from high false positive rates, but we show that this is unlikely to affect the results of Wham’s association testing. Wham’s ease of use also makes it an ideal package for inclusion with integrated SV callers. By simply running Wham in its default association-testing mode, we were able to identify the causal SV allele of a recessive trait in pigeons. Similarly, Wham’s accurate breakpoint predictions were able to locate a copy number variant in viral populations relative to a parental strain with very high precision.

## Availability and Future Directions

Wham and all associated software can be found on github (https://github.com/zeeev/wham), documentation is on the wiki (http://zeeev.github.io/wham/). For community support please post questions to https://Biostars.org [63].

## Supporting Information

S1 File Additional information regarding simulations, benchmarks, biological datasets, validations, and software versions. (DOCX)

S1 Table A description of the factors used in structural variant classification. These factors are found in the VCF info field under the “AT” key.
(DOCX)

S2 Table The number of structural variant calls for NA12878 before and after filtering. (DOCX)

S3 Table Breakpoint support and accuracy in the vaccinia virus dataset. (DOCX)

S1 Fig Wham false positives and true positives share similar p-value distributions. Quantile-quantile plots for Wham’s LRT statistic after conversion to p-values (y-axis). **Left panel:** The p-values for the structural variants that intersect with The 1000 Genomes Project Phase 3 dataset (within +/- 25 bp). **Right panel:** The p-values for structural variants that do not intersect with the phase III 1000 Genomes Project dataset. Both the true and false positive SV calls have very similar distributions.
(PDF)

S2 Fig Wham and Delly runtimes for a one Mb region across differing samples sizes. Wham has a linear relation between runtime and the number of samples run. The black line has a y-intercept of zero and slope of one.
(PDF)
