# Supplementary material for: Wham: Identifying Structural Variants of Biological Consequence
Source: PLoS Comput Biol. 2015 Dec 1;11(12):e1004572. doi: 10.1371/journal.pcbi.1004572 (PMC4666669; doi:10.1371/journal.pcbi.1004572)
Supplement: S1 File — (DOCX) [file pcbi.1004572.s001.docx]

## Simulations & Human Benchmarks

Non-overlapping homozygous deletions, duplications, inversions and translocations/insertions, were independently injected into the human reference genome (hg19, GATK resource bundle (DePristo et al., 2011; McKenna et al., 2010)) using SVsim (Faust). SVsim generates insertions by placing fragments from another chromosome into the target site; therefore we denote insertions as translocations/insertions. Two SV sizes, one and five, were incremented by powers of 10 (1-6) generating SVs from 50 bp to 1Mb. From the mutated sequences, 150-bp paired-end reads were simulated using DWGsim at two average depths, ten and fifty (Homer). DWGsim added single nucleotide polymorphisms, but no additional SVs. The simulated reads were aligned to the human reference genome using BWA mem in the default mode (Li, 2013). The alignments were converted to BAM files, sorted, and duplicates were removed using Samtools (Li et al., 2009). Each sample was run independently with Wham, Delly and Lumpy. Wham and Delly VCFs were converted to BEDPE format using two scripts distributed with Wham (whamToBedPe.pl and dellyToBedPe.pl). For benchmarking we used bedtools set operations to determine true and false positives (Quinlan and Hall, 2010). The ‘pairToPair –type both –slop 50 –is’ command was used to find true positives, requiring both putative breakpoints overlap with a single simulated SV. False positives were counted with ‘pairToPair –type notboth –slop 50 –is’. This benchmarking scheme has previously been used and honors the confidence intervals provided by all three tools. See the repository for the commands used for simulation.

All of Illumina’s platinum genomes (17 member CEPH pedigree 1463) were aligned using BWA mem, sorted with Sambamba (v0.5.0-dev), and duplicates were removed with Samblaster (0.1.20) (Faust and Hall, 2014; Tarasov). The truth set (phase III One Thousand Genomes Project) was downloaded from dbVar (<http://www.ncbi.nlm.nih.gov/news/11-04-2014-1000-genomes-phase-3-data-dbvar/>) and converted to BEDPE with 25 bp of bi-directional slop added. Both the truth and caller-derived SV sets were further filtered with the low complexity region file (Li, 2014) and the high coverage region file provided by Lumpy (Layer et al., 2014). These sequential filters were done with the ‘pairToBed -type neither’ command in bedtools. The benchmarks for NA12878 followed the same procedures as the simulation except for the SoftSearch benchmarks. We used ‘pairToPair –type either –slop 50 –is’ for true positives and ‘pairToPair –type neither –slop 50 –is’ for false positives as SoftSearch often failed to correctly identify both breakpoints.

The CHM1 publicly available structural variant call set (<http://eichlerlab.gs.washington.edu/publications/chm1-structural-variation>) was downloaded with the accompanying 101-bp Illumina reads (SRX533609). We converted the CHM1 bed files to BEDPE files adding 50 bp of bi-directional slop. Without slop, the concordance of the tools tested here and the CHM1 datasets was too low. The Illumina reads were aligned to the b37 (GATK resource bundle) reference genome using BWA mem version 0.7.10-r868-dirty (Li, 2013). The aligned reads were sorted and duplicates were removed using Samtools version 0.1.19-44428cd (Li et al., 2009). Wham, Lumpy and Delly were run in default mode over the Illumina BAM files. The benchmarks used the same methods as previously described without the filtering steps as many of the CHM1 calls are in low complexity regions.

## Biological datasets

All biological datasets are publically available on Sequence Reads Archive (SRA). The eleven re-sequenced pigeons used in the association study can be found under SRA054391. The five recessive red birds have the following SRA ids: SRS346872, SRS346882, SRS346899, SRS346902, SRS346883 and the non-recessive red birds (backgrounds) have the following SRA ids: SRS346895, SRS346873, SRS346870, SRS346896, SRS346874, SRS346877. Each re-sequenced bird has a depth of coverage ~10x from paired-end Illumina reads. The reads were aligned to the pigeon assembly (C_liv1.0, GenBank assembly accession: GCA_000337935.1). The viral dataset, including the parental (SRS812401) and adapted strain (SRS812403), are associated with SRP051821.

Wham was run over both the viral and pigeon datasets in default mode. In the pigeon genome-wide association study, we removed sites where there were no-calls (missing genotypes). Sites with LRT values less than 1.5 were also excluded for the Manhattan plot for the purpose of visualizing the dataset. We also removed sites with less than three reads supporting the start position. In the VACV dataset we removed inverted terminal repeats (10 kb on either end of the genome) to avoid false positives from reads mapping to complementary regions of the genome. We then excluded sites where the start position was supported by fewer than 50 reads. Similarly we discarded Lumpy calls that had fewer than 50 reads supporting a SV. Increasing the filter to 100 removed almost all of the spurious calls from Lumpy.

The genotype-phenotype association test for the *e1* allele using Delly data was done using GPAT++ (Kronenberg). Wham’s likelihood ratio test is implemented in pFst using the “count” setting. Delly was run over the pigeon BAM files using all four modes (DEL, DUP, INV, TRA). These different call sets were merged (union) and passed to pFst (Kronenberg).

## PCR validation in the poxvirus dataset

The ΔE3L vaccinia virus was passaged 10 times in primary human fibroblasts at a multiplicity of infection of 0.1 for 48 hours (see [41] for details). Deep sequencing of viral populations was performed on libraries prepared from genomic viral DNA isolated from either the parental ΔE3L or an adapted strain after 10 serial passages using the Nextera XT DNA sample prep kit (Illumina, Inc., San Diego, CA, USA). Barcoded libraries were combined and sequenced on a single lane using an Illumina MiSeq instrument. Reads were mapped to the vaccinia virus Copenhagen strain reference genome (accession M35027.1; modified on poxvirus.org) using BWA mem in default mode, duplications were removed using samtools, and SVs in the adapted and parental strains were called with both Wham and Lumpy.

PCR primers were designed to amplify products across the potential breakpoints identified by Wham. The K3L breakpoint was amplified using primers K3L break F (5’ GGGATAAACTGGTAGGGAAAACTGTAAAAG 3’) and K3L break R (5’ CAGAGTGAGGATAGTCAAAAAGATAAATGTATAG 3’). The E3L deletion junctions were amplified using E2L int F (5’ GGAGCTACAGTTCTTGGC 3’), E4L int R (5’ CCTTCGCTATCTCTTATTCGG 3’), and 46731R (5’ CTAGCGTACGATCGCTTCTAG 3’). The resulting products were Sanger sequenced and aligned to the reference genome using blastn (NCBI).

## Software versions:

Wham: v1.7.0-109-g1eed-dirty

Lumpy: v 0.2.11

Delly: Version: 0.6.6

SoftSearch: Version 2.4

Svtyper: v0.0.1-79-g04bcf70

## Supplement references

DePristo, M.A., Banks, E., Poplin, R., Garimella, K. V, Maguire, J.R., Hartl, C., Philippakis, A.A., del Angel, G., Rivas, M.A., Hanna, M., et al. (2011). A framework for variation discovery and genotyping using next-generation DNA sequencing data. Nat. Genet. *43*, 491–498.

Faust, G. SVsim. https://github.com/GregoryFaust/SVsim

Faust, G.G., and Hall, I.M. (2014). SAMBLASTER: fast duplicate marking and structural variant read extraction. Bioinformatics *30*, 2503–2505.

Homer, N. DWGSIM. https://github.com/nh13/DWGSIM

Kronenberg, Z. GPAT++. https://github.com/jewmanchue/wham/wiki

Layer, R.M., Chiang, C., Quinlan, A.R., and Hall, I.M. (2014). LUMPY: A probabilistic framework for structural variant discovery. Genome Biol. *15*, R84.

Li, H. (2013). Aligning sequence reads, clone sequences and assembly contigs with BWA-MEM. arXiv Prepr. arXiv1303.3997 *00*, 1–3.

Li, H. (2014). Towards better understanding of artifacts in variant calling from high-coverage samples. Bioinformatics 1–8.

Li, H., Handsaker, B., Wysoker, A., Fennell, T., Ruan, J., Homer, N., Marth, G., Abecasis, G., Durbin, R., and others (2009). The sequence alignment/map format and SAMtools. Bioinformatics *25*, 2078–2079.

McKenna, A., Hanna, M., Banks, E., Sivachenko, A., Cibulskis, K., Kernytsky, A., Garimella, K., Altshuler, D., Gabriel, S., Daly, M., et al. (2010). The Genome Analysis Toolkit: a MapReduce framework for analyzing next-generation DNA sequencing data. Genome Res. *20*, 1297–1303.

Quinlan, A.R., and Hall, I.M. (2010). BEDTools: a flexible suite of utilities for comparing genomic features. Bioinformatics *26*, 841–842.

Tarasov, A. sambamba. https://github.com/lomereiter/sambamba
