# Supplementary material for: Wham: Identifying Structural Variants of Biological Consequence
Source: PLoS Comput Biol. 2015 Dec 1;11(12):e1004572. doi: 10.1371/journal.pcbi.1004572 (PMC4666669; doi:10.1371/journal.pcbi.1004572)
Supplement: S1 Table — These factors are found in the VCF info field under the “AT” key. (DOCX) [file pcbi.1004572.s002.docx]

| **Category** | **Notes** | **Importance** |
| --- | --- | --- |
| Both mates mapped | NOT USED | NOT USED |
| 1. Discordant | Not paired | 0.056 |
| 2. Mate not mapped | SAM bit flag 8 | 0.0 |
| 3. Mates mapped to same strand | Mate pairs on same strand | 0.089 |
| 4. Mates on different seqids | RefID != MateRefID | 0.148 |
| 5. Number of split reads | Contains the ‘SA’ optional tag | 0.078 |
| 6. Split read (fragment 1) on same strand as mate | Combination of SAM flag and SA tag | 0.071 |
| 7. Split read (fragment 2) on same strand as mate | Combination of SAM flag and SA tag | 0.071 |
| 8. Split read (fragment 1) and read two (fragment 2) on same strand | Combination of SAM flag and SA tag | 0.13 |
| 9. Internal insertion | Cigar string contains ‘I’ operation | 0.0002 |
| 10. Internal deletion | Cigar string contains ‘D’ operation | 0.0135 |
| 11. Mates mapped too close | Insert < (2.5 * sd average insert size) | 0.024 |
| 12. Mates mapped to far | Insert > (2.5 * sd average insert size) | 0.101 |
| 13. Everted pairs | Position and orientation of mate pairs | 0.075 |
| 14. Relative depth | Depth at position for each sample relative to their mean depth | 0.137 |

### Table S1. The factors used to classify the SV type.

All factors reported in the VCF “AT” info field are the fraction of the reads in the pileup falling into one or more of the 14 categories described above. During the training step, the importance of each factor is reported as shown above. The importance is the relative weight each attribute contributes to the classification.
