# Supplementary material for: Wham: Identifying Structural Variants of Biological Consequence
Source: PLoS Comput Biol. 2015 Dec 1;11(12):e1004572. doi: 10.1371/journal.pcbi.1004572 (PMC4666669; doi:10.1371/journal.pcbi.1004572)
Supplement: S2 Table — (DOCX) [file pcbi.1004572.s003.docx]

|  | Delly | LUMPY | SS | WHAM | Phase III NA12878 |
| --- | --- | --- | --- | --- | --- |
| Total calls | 96,583 | 7,955 | 52,394 | 348,761 | 2,597 |
| LowQual | 5,058 | NA | NA | NA | NA |
| LCR filter | 2,174 | 3,848 | 30,270 | 90,911 | 2,202 |
| High coverage filter | 2,174 | 3,620 | 25,281 | 84,422 | 2,146 |
| Deletion only | 1,978 | 2,120 | 8,066 | 3,891 | 688 |

### Table S2. SV calls remaining after each filtering step

The total number of SV calls (of any type) for each tool is listed in the first row. The second row is a Delly specific filter, removing SVs with the “LowQual” tag. The low complexity region (LCR) filter removes regions that are difficult to map in (see Simulations & Human Benchmarks in Supporting Information). The high coverage filter removes sites displaying abnormally high read depths. The last row is the number of deletions called by each tool.
