# Supplementary material for: Wham: Identifying Structural Variants of Biological Consequence
Source: PLoS Comput Biol. 2015 Dec 1;11(12):e1004572. doi: 10.1371/journal.pcbi.1004572 (PMC4666669; doi:10.1371/journal.pcbi.1004572)
Supplement: S3 Table — (DOCX) [file pcbi.1004572.s004.docx]

| Wham breakpoint | Wham  breakpoint position | WHAM  SR count | WHAM  MP count | Lumpy  breakpoint position |
| --- | --- | --- | --- | --- |
| 1 | 31725* | 977 | 73 | 31725* |
| 2 | 30296* | 456 | 100 | 30295 |
| 3 | 50913* | 0 | 6 | N/A |
| 4 | 46840 | 1 | 160 | 46746 |
| 5 | 46731* | 344 | 65 | N/A |
| 6 | 51483* | 314 | 62 | 51482 |

### Table S3. Wham and Lumpy breakpoint positions in the adapted vaccinia virus population (related to Fig 4)

Wham and Lumpy breakpoint positions in the adapted vaccinia virus genome, corresponding to the breakpoints shown in Fig 4. WHAM split-read (SR) and mate-pair (MP) read support is listed for each position. Asterisks (*) indicate breakpoints for which the breakpoint position is the same as the Sanger sequencing verified breakpoint.
