# Supplementary figures and images for: Wham: Identifying Structural Variants of Biological Consequence
Source: PLoS Comput Biol. 2015 Dec 1;11(12):e1004572. doi: 10.1371/journal.pcbi.1004572 (PMC4666669; doi:10.1371/journal.pcbi.1004572)

**true positive SVs**

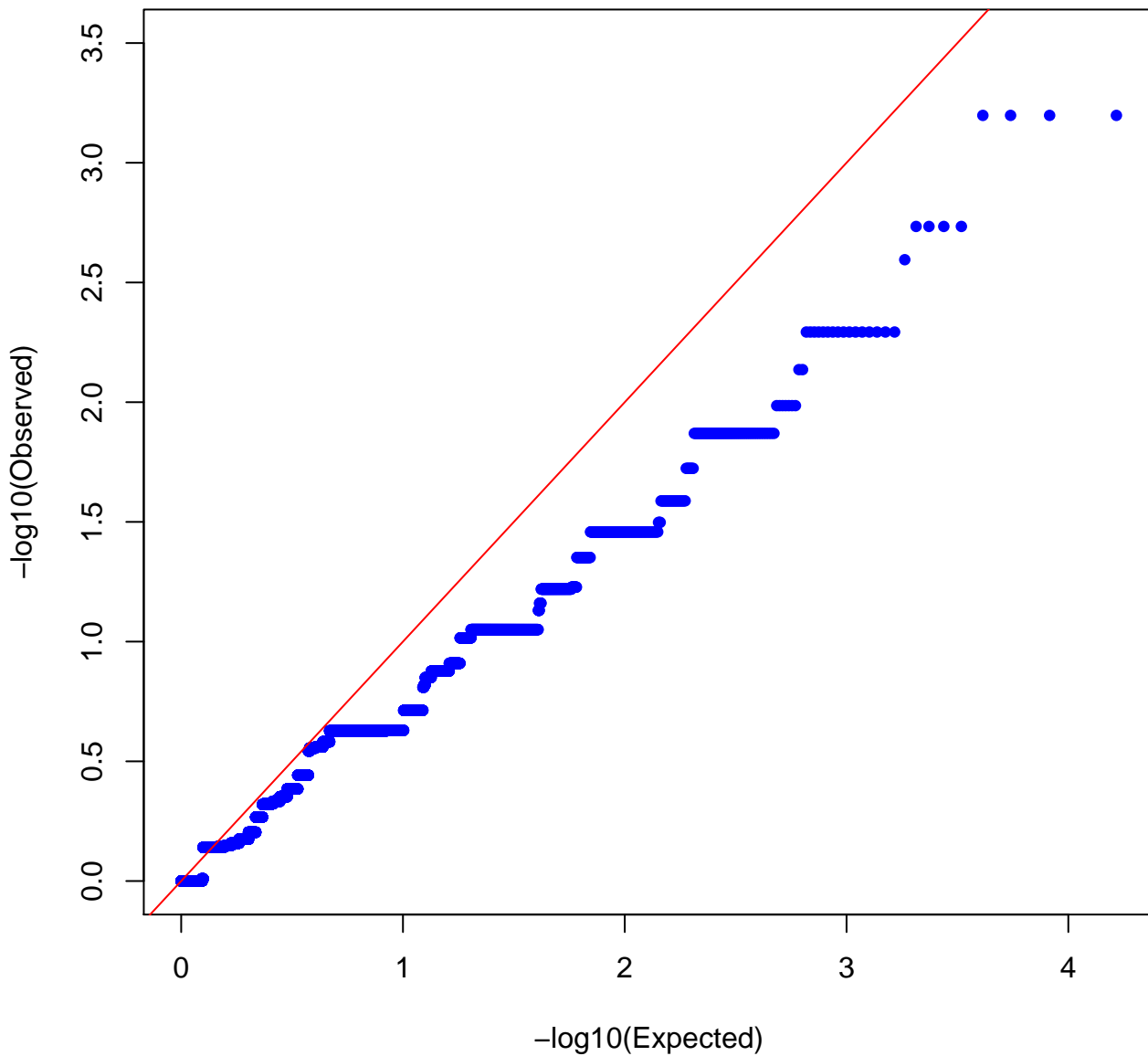

**false positive SVs**

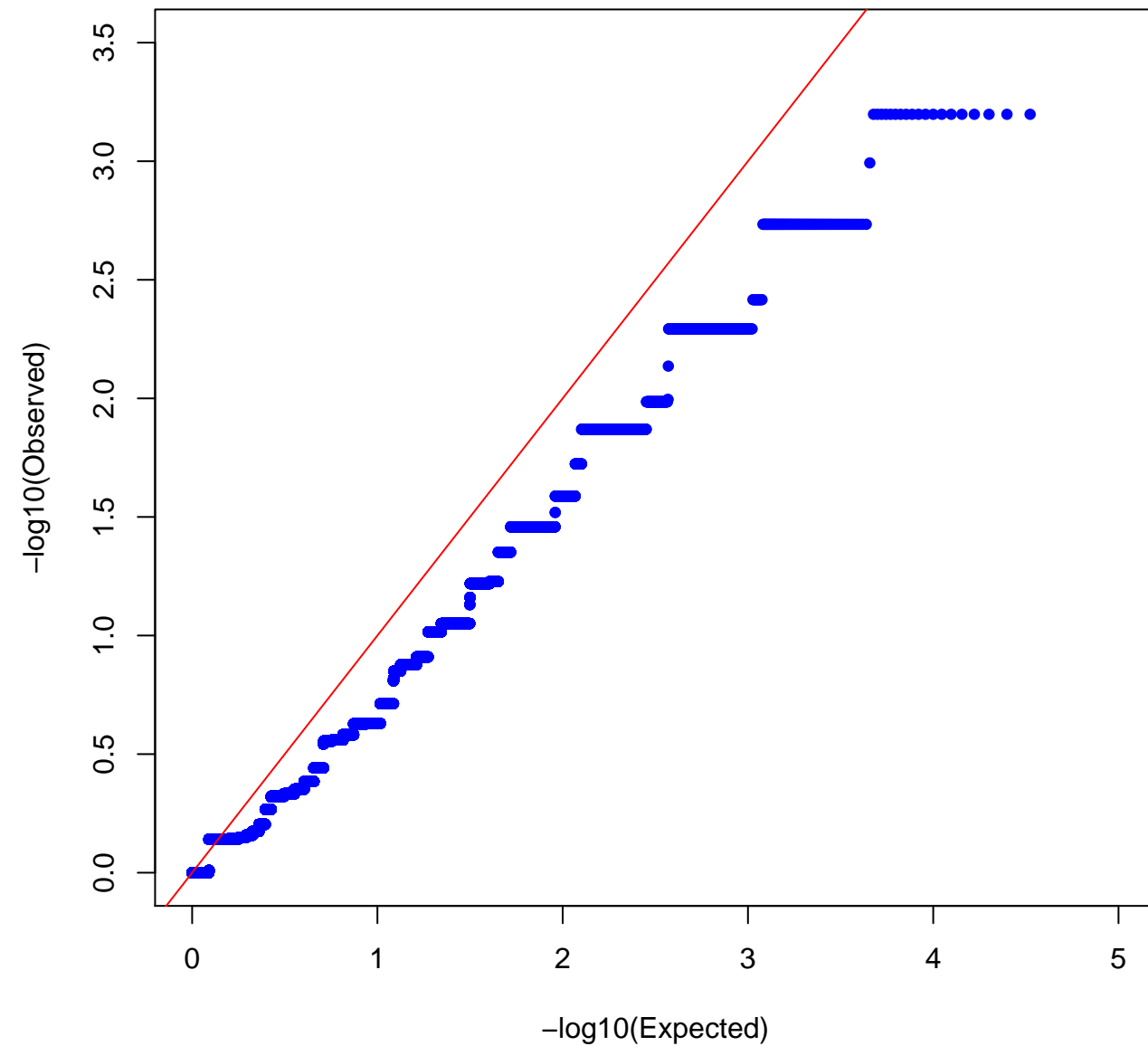

Supplement: S1 Fig — Quantile-quantile plots for Wham’s LRT statistic after conversion to p-values (y-axis). Left panel: The p-values for the structural variants that intersect with The 1000 Genomes Project Phase 3 dataset (within +/- 25 bp). Right panel: The p-values for structural variants that do not intersect with the phase III 1000 Genomes Project dataset. Both the true and false positive SV calls have very similar distributions. (PDF) [file pcbi.1004572.s005.pdf]

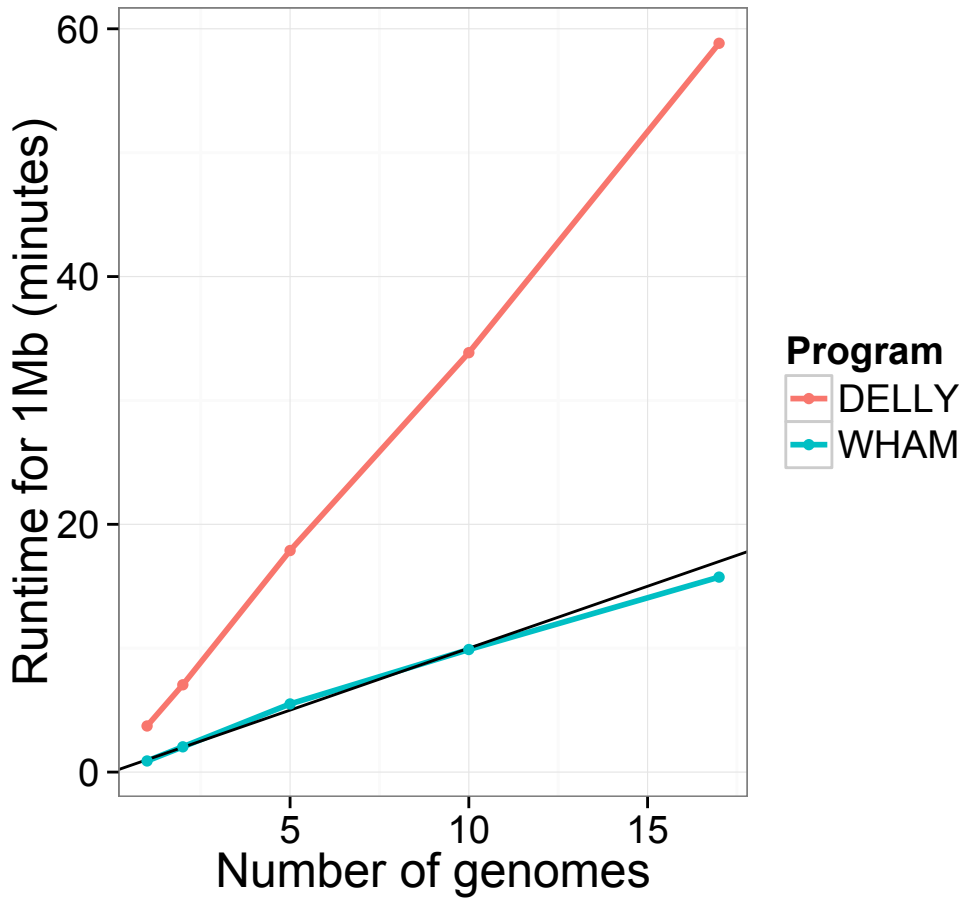

Supplement: S2 Fig — Wham has a linear relation between runtime and the number of samples run. The black line has a y-intercept of zero and slope of one. (PDF) [file pcbi.1004572.s006.pdf]
